# Transcriptomic Analysis of Octanoic Acid Response in *Drosophila sechellia* Using RNA-Sequencing

**DOI:** 10.1534/g3.117.300297

**Published:** 2017-10-11

**Authors:** Stephen M. Lanno, Sara M. Gregory, Serena J. Shimshak, Maximilian K. Alverson, Kenneth Chiu, Arden L. Feil, Morgan G. Findley, Taylor E. Forman, Julia T. Gordon, Josephine Ho, Joanna L. Krupp, Ivy Lam, Josh Lane, Samuel C. Linde, Ashley E. Morse, Serena Rusk, Robie Ryan, Avva Saniee, Ruchi B. Sheth, Jennifer J. Siranosian, Lalitpatr Sirichantaropart, Sonya R. Sternlieb, Christina M. Zaccardi, Joseph D. Coolon

**Affiliations:** Department of Biology, Wesleyan University, Middletown, Connecticut 06457

**Keywords:** host specialization, octanoic acid, toxin resistance, gene expression, RNA-seq

## Abstract

The dietary specialist fruit fly *Drosophila sechellia* has evolved to specialize on the toxic fruit of its host plant *Morinda citrifolia*. Toxicity of *Morinda* fruit is primarily due to high levels of octanoic acid (OA). Using RNA interference (RNAi), prior work found that knockdown of *Osiris* family genes *Osiris 6* (*Osi6*), *Osi7*, and *Osi8* led to increased susceptibility to OA in adult *D. melanogaster* flies, likely representing genes underlying a Quantitative Trait Locus (QTL) for OA resistance in *D. sechellia*. While genes in this major effect locus are beginning to be revealed, prior work has shown at least five regions of the genome contribute to OA resistance. Here, we identify new candidate OA resistance genes by performing differential gene expression analysis using RNA-sequencing (RNA-seq) on control and OA-exposed *D. sechellia* flies. We found 104 significantly differentially expressed genes with annotated orthologs in *D. melanogaster*, including six *Osiris* gene family members, consistent with previous functional studies and gene expression analyses. Gene ontology (GO) term enrichment showed significant enrichment for cuticle development in upregulated genes and significant enrichment of immune and defense responses in downregulated genes, suggesting important aspects of the physiology of *D. sechellia* that may play a role in OA resistance. In addition, we identified five candidate OA resistance genes that potentially underlie QTL peaks outside of the major effect region, representing promising new candidate genes for future functional studies.

Most species of insects are plant feeding and have specialized to eat a small number of closely related species (Eastop 1973; Price *et al.* 1980; Mitchell 1981; Ehrlich and Murphy 1988; Jolivet 1992; Bernays and Chapman 1994). This leads to an arms race as plants make chemicals to deter herbivory and insects evolve behavioral, morphological, and physiological adaptations to facilitate resistance to their novel hosts (Jaenike 1987; Via 1990; Futuyma 1991). Ecological adaptation of insects to new host plants is well-documented, but the underlying genetic mechanisms governing these adaptations are much less understood. The Seychelles islands endemic fruit fly species *Drosophila sechellia* is an excellent model system for exploring the genetic basis of such ecological specialization. Host specialization in *D. sechellia* is intriguing, as the evolution of several recently acquired adaptive traits has allowed this species to feed solely on a host plant that is toxic to other Drosophilids: *Morinda citrifolia* (Louis and David 1986; R’Kha *et al.* 1991; Legal *et al.* 1992; Matute and Ayroles 2014). The ripe fruit of *M. citrifolia* contains high levels of octanoic acid (OA), a medium chain fatty acid that *D. sechellia* has evolved resistance to and preference for, and is largely responsible for the fruit’s toxicity (Legal *et al.* 1994; Farine *et al.* 1996; Amlou *et al.* 1997). Species in the *D. melanogaster* supercomplex are well positioned for examining the genetics of host specialization as this clade contains OA-susceptible generalist species *D. melanogaster*, *D. simulans*, and *D. mauritiana*, and the single derived resistant specialist species nested within this group, *D. sechellia*. This relationship allows for the analysis of gene expression and coding sequence divergence among members of this clade, as well as functional gene testing using the numerous genetic and genomic tools available in the model organism *D. melanogaster*.

Relatively few studies have focused on identifying the genetic factors that confer resistance to specific toxic compounds in host specialist insect species. While the genetics of OA resistance in *D. sechellia* is one of the best researched case studies, the specific genes involved in OA resistance are just beginning to be revealed. Using genetic markers, initial mapping data of OA resistance factors were determined in *D. sechellia* adults (Jones 1998) and later in *D. sechellia* larvae (Jones 2001). In adults, at least five loci were found to be involved in *D. sechellia* OA resistance. Two factors on the X chromosome, one factor of weak effect on chromosome 2, and two resistance factors on the third chromosome, with a region of major effect on chromosome 3R that explains ∼15% of the variation in OA resistance between *D. sechellia* and susceptible sister species *D. simulans*, while representing only 2–3% of the *D. sechellia* genome (Jones 1998, 2005). While mapping data of *D. sechellia* larval OA resistance regions has been greatly improved in the last few years due to the use of hundreds of thousands of genetic markers (Huang and Erezyilmaz 2015), adult resistance regions primarily involve QTL analyses with C.I.s that are too large to yield specific candidate genes. However, the region of greatest effect on adult OA resistance on chromosome 3R has been fine-mapped (Hungate *et al.* 2013).

Fine-mapping narrowed the largest effect resistance region to a ∼170 kb window containing 18 genes, including several odorant binding proteins (*Obps*) and nine members of the *Osiris* gene family (Hungate *et al.* 2013). These 18 genes were functionally tested using RNAi in *D. melanogaster* adults to identify candidate OA resistance genes (Andrade López *et al.* 2017). Three genes in the *Osiris* family, *Osiris 6* (*Osi6*), *Osi7*, and *Osi8*, were found to significantly decrease OA resistance when their expression was ubiquitously knocked down. Corresponding gene expression analyses revealed that *Osi6* and *Osi7* were expressed significantly higher in *D. sechellia* than *D. simulans*, matching the RNAi result in *D. melanogaster*, suggesting these genes may play an important role in *D. sechellia* OA resistance. *Osiris 8* did not show differential expression between these species. However, two protein-coding sequence changes observed in the *D. sechellia* allele of *Osi8* suggests altered protein function may be involved in *Osi8*-mediated OA resistance (Andrade López *et al.* 2017; S.M. Lanno, unpublished results). Yassin *et al.* (2016) found further support for *Osiris* gene involvement in OA resistance and showed that the *Osiris* gene cluster was among the strongest differentiation peaks in a population genomics scan between an island specialist population of *D. yakuba*, which recently specialized on *M. citrifolia*, and a mainland generalist population of the same species.

To begin to identify the specific gene(s) underlying OA resistance in *D. sechellia*, we used genome-wide gene expression profiling in response to OA exposure, because prior work has shown that genes identified as differentially expressed between environments contribute to fitness differences between those environments (Coolon *et al.* 2009). Therefore, identification of patterns in gene expression plasticity in response to OA is important the determination of the mechanism of toxin resistance critical to host specialization. In this study, we reared adult female *D. sechellia* flies on control food and food containing 0.7% OA, and identified genes that are differentially expressed as a result of exposure to OA that fall into QTL mapped regions, providing a set of excellent candidates for future functional investigation.

## Materials and Methods

### Fly strain and maintenance

**Figure 1:**
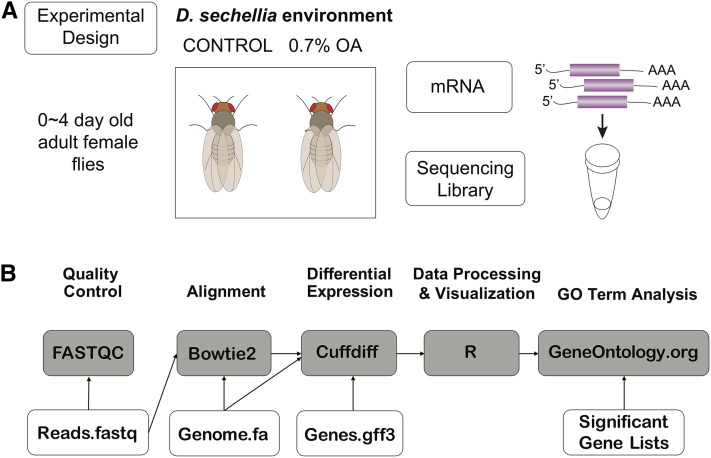
Experimental design and RNA-sequencing (RNA-seq) pipeline. (A) *D. sechellia* flies were reared on cornmeal medium. Adult female flies, 0–4 d old, were exposed to either control food or food containing 0.7% octanoic acid (OA). Total RNA was extracted from replicates of 10 whole adult female flies. (B) RNA-seq pipeline performed in Galaxy. Read quality was checked using FASTQC and aligned to the *D. sechellia* genome using Bowtie2. Differential gene expression statistical analysis was performed with Cuffdiff. All subsequent processing and visualization was performed in R and gene ontology (GO) term enrichment was performed using the Gene Ontology Consortium.

*D. sechellia* (14021-0428.25) flies were reared on cornmeal medium using a 16:8 light:dark cycle at 20°. Adult females that were 0–3 d old were collected and exposed to either control food (Carolina Biological Supply Company), or food containing 0.7% OA (Sigma) for an exposure period of 24 hr (Figure 1A). Following the exposure period, flies were snap-frozen in liquid nitrogen and stored at −80° until RNA extraction. We used 0.7% OA mixed into food for exposure of the *D. sechellia* adult flies because this concentration of OA mimics the high end of the natural concentration of OA in fresh *Morinda* fruit, as evidenced by studies of behavioral response to OA as compared to fresh *Morinda* fruit (Amlou *et al.* 1998), chemical analyses of fresh *Morinda* fruit (Farine *et al.* 1996), and the fact that 0.7% OA in the food did not elicit any mortality in adult *D. sechellia*.

### RNA extraction, library prep, and RNA-seq

RNA was extracted from a homogenate of 10 whole adult flies using the Promega SV total RNA extraction system with modified protocol (Promega; Coolon *et al.* 2013). Three replicates were analyzed for each exposure environment (control or 0.7% OA). A total of six RNA-seq samples were prepared by poly(A) selection. RNA quality control was performed by gel electrophoresis and NanoDrop to confirm successful RNA extraction, quantify concentration, and check for sample degradation. RNA samples were sent to the University of Michigan Medical School DNA Sequencing Core, where bar-coded library preparation was done using TruSeq library preparation kits and uniform library abundance in a pool of 21 samples was confirmed with qPCR. Then, the pooled barcoded samples were sequenced in one lane using an Illumina HiSeq-4000 generating single-end 50 nt sequence reads.

### RNA-seq analysis and pipeline

The bioinformatics analysis reported in this manuscript was performed by a group of 20 undergraduate students and three Master’s students as part of a course at Wesleyan University called Genomics Analysis (BIOL310). After obtaining sequence read files, an RNA-seq pipeline was performed in Galaxy (Afgan *et al.* 2016) (Figure 1B). Raw sequencing reads were checked for quality using FASTQC (Andrews 2010) and overrepresented sequences were analyzed using NCBI BLAST (Altschul *et al.* 1990). Reads were mapped to the *D. sechellia* genome using Bowtie2 with default parameters (Langmead and Salzberg 2012) and the current genome file available at the time analysis from Ensembl: Drosophila_sechellia.GCA_000005215.1.dna.toplevel.fa (Yates *et al.* 2016). Gene expression quantification and differential expression analysis was performed with Cuffdiff (Trapnell *et al.* 2010, 2013) using the previously mentioned genome file and the current gene file available from Ensembl: Drosophila_sechellia.GCA_000005215.1.34.gff3 (Yates *et al.* 2016). In Cuffdiff, geometric normalization and length correction was used and bias correction was performed using the reference genome sequence. Data processing and visualization was performed in R (R Core Development Team 2010). *D. melanogaster* gene name orthologs were downloaded from FlyBase (Attrill *et al.* 2016) for all *D. sechellia* genes for use in GO term enrichment analysis. GO term enrichment analysis and visualization was performed on all significantly differentially expressed genes using the Gene Ontology Consortium online tool (The Gene Ontology Consortium 2000, The Gene Ontology Consortium 2015, www.geneontology.org).

### OA resistance assay

Adult female *D. simulans* flies that were 0–4 d old (14021-0251.195) were collected from bottles containing cornmeal medium. Six replicates of 10 female adult flies were placed into vials containing 0.75 g *Drosophila* medium (Carolina Biological Supply Company Formula 4–24), 2.5 ml H_2_O, and 22.75 µl 99% OA (AC129390010; Fisher Scientific) (0.7% OA by weight). The number of flies “knocked down” was determined every 5 min for a period of 60 min. A fly was counted as knocked down when the individual was not able to walk or fly [as described in Andrade López *et al.* (2017)].

### Data accessibility

The sequencing data from this study have been submitted to the NCBI Gene Expression Omnibus (http://www.ncbi.nlm.nih.gov/geo), accession number: GSE104422.

## Results

### RNA-seq

**Figure 2:**
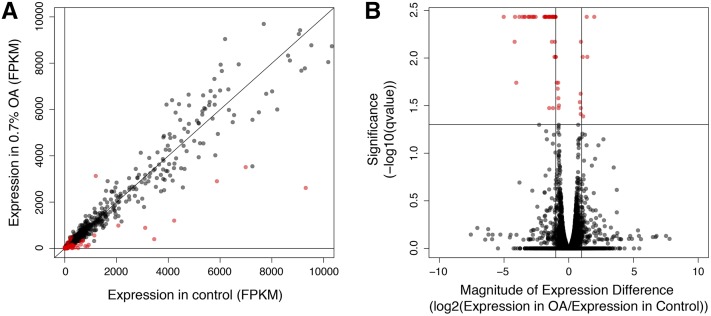
RNA-sequencing analysis of *D. sechellia* gene expression response to 0.7% OA. (A) Scatterplot of all differentially expressed genes in *D. sechellia* adult female flies upon exposure to 0.7% octanoic acid (OA) in Fragments Per Kilobase of transcript per Million mapped reads (FPKM) is shown (red = significant, black = nonsignificant). (B) Volcano plot of differentially expressed genes plotting the magnitude of expression difference against significance from the statistical test performed. The horizontal line represents *q*-value = 0.05.

To investigate the transcriptional response of *D. sechellia* to OA exposure, we measured genome-wide gene expression with RNA-seq. This produced an average of 20,420,699 reads across six sequencing libraries, ranging from ∼17.7 to 26.6 million reads per sample (Table 1). One-hundred thirty-two unique genes showed significant differential expression (FDR < 0.05) between the control and OA-exposed treatment groups, 104 of which have annotated orthologs in *D. melanogaster* and were used for the remainder of the analysis in this study (Supplemental Material, Table S1 in File S1). The 28 genes lost in this conversion include three 5S rRNA (RF) genes, four snoRNAs, five snRNAs, six 18S rRNAs, and 10 genes that have no annotated ortholog in *D. melanogaster* (full descriptions of these genes are in Table S2 in File S1). Of the 104 significantly differentially expressed genes (Figure 2), significantly more were downregulated (64/104) than were upregulated (40/104) in response to OA (Binomial Exact Test, *P* = 0.024). Significantly upregulated genes included six members of the *Osiris* gene family, *Osi6*, *Osi9*, *Osi15*, *Osi18*, *Osi19*, and *Osi20* (Table 2), and five members of the *Tweedle* gene family, *TwdlG*, *TwdlL*, *TwdlM*, *TwdlV*, and *TwdlY*. Significantly downregulated genes included genes related to immune and defense responses, and chorion proteins, such as *AttA*, *AttC*, *Cp16*, *Cp18*, *Cp19*, *Def*, *edin*, *IM2*, *IM18*, *IM23*, *Peritrophin-15a*, and *term*.

**Table 1:** Total number of mapped reads for each sequencing library

Sample	# Reads	# Mapped Reads	% Mapped
Control Rep 1 (76332)	19,222,060	18,496,450	96.23
Control Rep 2 (76333)	20,704,811	19,440,620	93.89
Control Rep 3 (76334)	17,696,868	17,123,579	96.76
0.7% OA Rep 1 (76335)	20,398,289	18,355,454	89.99
0.7% OA Rep 2 (76336)	26,635,410	24,940,753	93.64
0.7% OA Rep 3 (76337)	17,866,761	16,851,323	94.32

Rep, replicate; OA, octanoic acid.

**Table 2:** Significantly differentially expressed *Osiris* genes upon exposure to 0.7% OA

*D. sechellia* Gene	*D. melanogaster* Ortholog	Expression in Control (FPKM)	Expression in OA (FPKM)	*q*-Value
GM10867	*Osi6*	0	3.026	0.0037
GM10870	*Osi9*	0	2.28	0.0037
GM10877	*Osi15*	0	9.91	0.0037
GM10882	*Osi18*	0	3.00	0.0037
GM10883	*Osi19*	0	5.02	0.0037
GM10884	*Osi20*	0	9.19	0.0037

FPKM, Fragments Per Kilobase of transcript per Million mapped reads; OA, octanoic acid.

### OA resistance-associated mapping data and QTL peaks

Genes involved in OA resistance may act through regulatory changes in gene expression, protein-coding sequence changes, or both. For genes with expression responses to OA exposure to contribute to OA resistance in *D. sechellia*, the response should be unique to *D. sechellia*. Because sister species *D. simulans* cannot survive exposure to the concentration of OA used in this study (Figure S1 in File S1), we can be sure that these genes are not differentially expressed in *D. simulans* in response to the same treatment and are therefore unique to *D. sechellia*.

**Figure 3:**
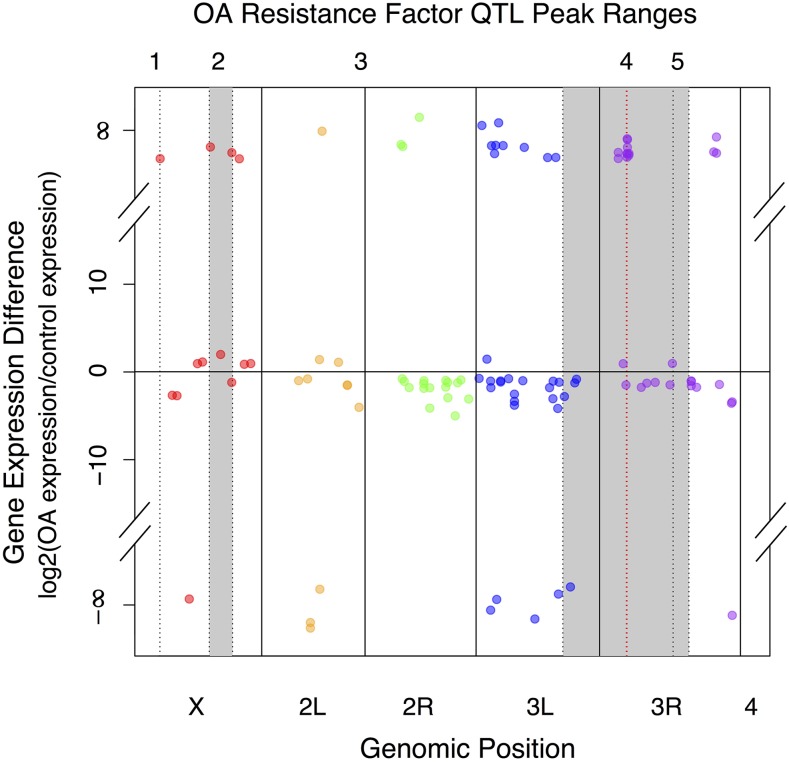
Significantly differentially expressed genes overlaid onto Quantitative Trait Locus (QTL) peak ranges for octanoic acid (OA) resistance in *D. sechellia* adults. Significantly differentially expressed genes are plotted according to chromosomal position (red = genes on the X chromosome, orange = 2L, green = 2R, blue = 3L, and purple = 3R). No significantly differentially expressed genes were found on the fourth chromosome. Shaded gray areas represent QTL peak ranges for adult *D. sechellia* OA resistance described by Jones (1998, 2005). Red dotted line represents the fine-mapped QTL region from Hungate *et al.* (2013).

By cross-referencing our RNA-seq data with previously published mapping data for OA resistance regions in *D. sechellia*, we were able to identify candidate OA resistance genes that may act through changes in gene expression, which will be the subject of future analyses. In Jones (1998), adult *D. sechellia* mapping data revealed at least five OA resistance factors to be present in the genome (Jones 1998). We plotted significantly differentially expressed genes by their expression level and position in the genome (Figure 3). Previously published OA resistance mapping data revealed QTL peaks on the X chromosome (1) near recombination map *1*-0, (2) one between *1*-35 and *1*-56, at least one resistance factor, (3) on chromosome 2 (however, the effect was too weak to allow further dissection of the region), two resistance factors on chromosome 3, (4) one between *3*-46.3 and *3*-59.4, and (5) one between *3*-59.4 and *3*-68.6 (Jones 1998, 2005). Our analysis revealed one gene (*l*(*1*)*sc*) near the *1*-0 resistance factor on the X chromosome, four genes that fall beneath the next X chromosome QTL (*CG15740*, *CG1368*, *CG9672*, and *TwdlY*), and one gene (*Osi6*) that falls within the fine-mapped resistance region on chromosome 3R, which has been previously shown to be differentially expressed between *D. sechellia* and susceptible sister species *D. simulans* in whole animals, and also significantly downregulated in a tissue-specific manner in *D. sechellia* salivary glands, both of which match resistance phenotypes in *D. melanogaster* RNAi studies (Andrade López *et al.* 2017) (Table 3). While the resistance region on chromosome 2 was too weak for further genetic mapping, our analysis revealed 30 significantly differentially expressed genes on this chromosome, with 11 genes on chromosome 2L and 19 genes on chromosome 2R.

**Table 3:** Significantly differentially expressed genes that fall within mapped OA resistance regions on chromosome X and 3R

*D. sechellia* Gene	*D. melanogaster* Ortholog	Expression in control (FPKM)	Expression in OA (FPKM)	OA Resistance QTL Region
GM19063	*l*(*1*)*sc*	0	2.048	X
GM13042	*CG15740*	0	5.15	X
GM17654	*CG1368*	6.80	26.66	X
GM13371	*CG9672*	49.12	21.16	X
GM13463	*TwdlY*	0	3.27	X
GM10867	*Osi6*	0	3.03	3R

FPKM, Fragments Per Kilobase of transcript per Million mapped reads; OA, octanoic acid; QTL, quantitative trait locus.

### GO term enrichment analysis

**Figure 4:**
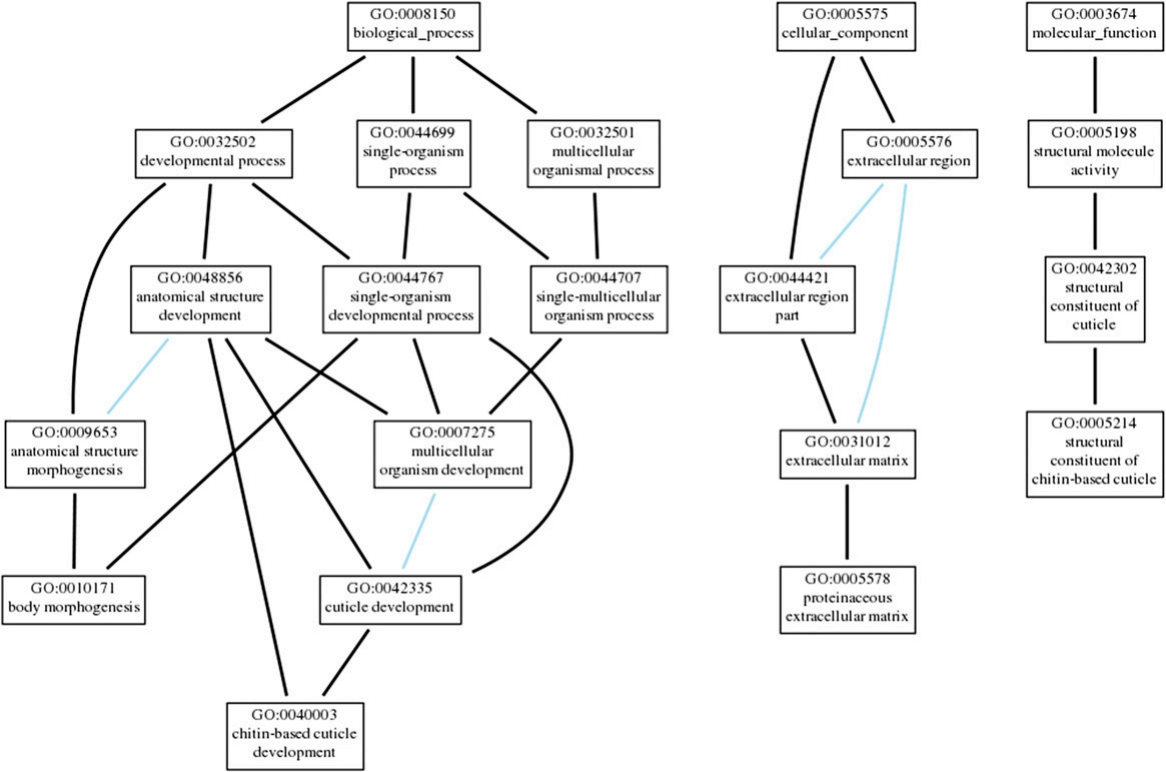
Gene ontology (GO) term enrichment for significantly upregulated genes. Visualization of GO terms associated with significantly upregulated genes in response to 0.7% octanoic acid. Each box contains the GO term identifier and description. Lines connecting GO terms represent ontology relationship (black lines indicate a regulatory relationship, and light blue lines indicate GO terms are a part of the term they connect to).

**Figure 5:**
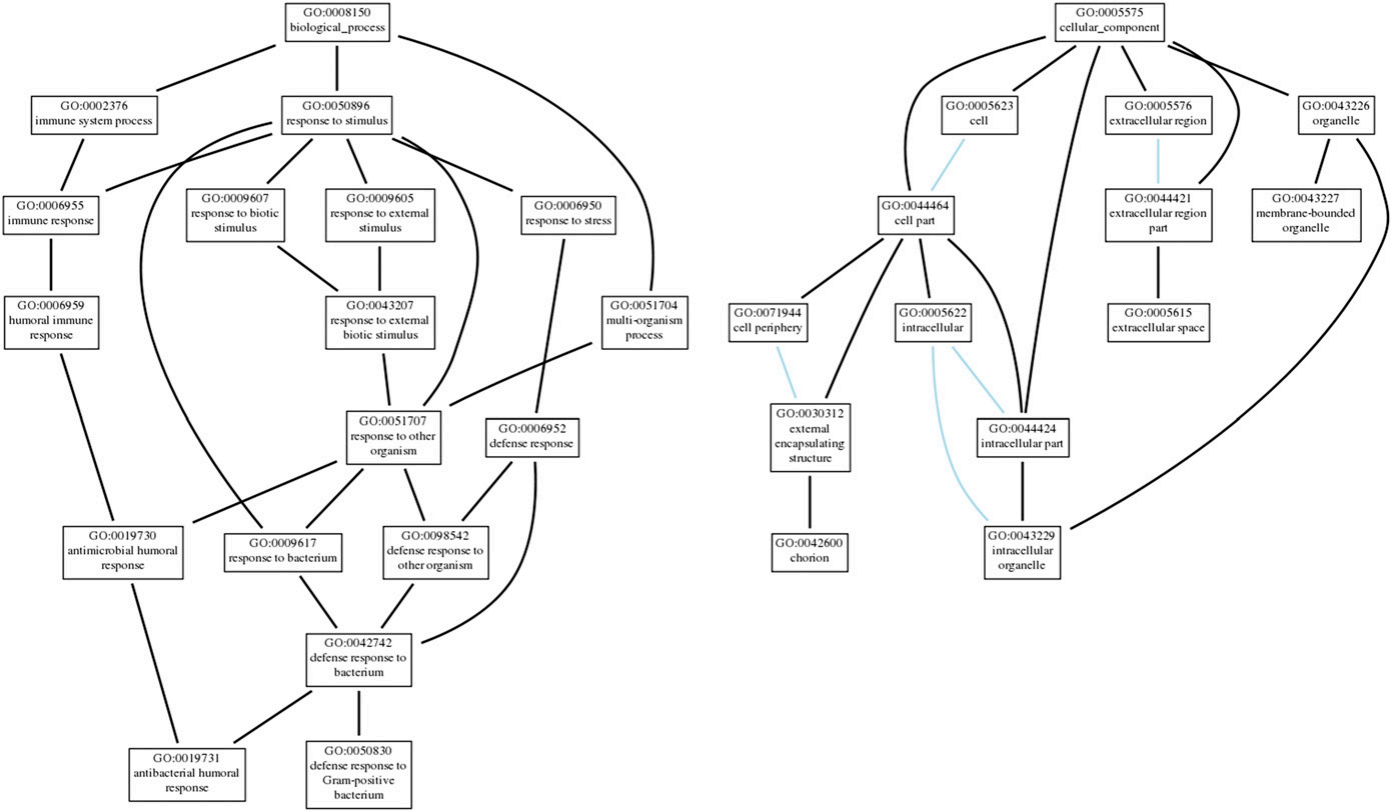
Gene ontology (GO) term enrichment for significantly downregulated genes. Visualization of GO terms associated with significantly downregulated genes in response to 0.7% octanoic acid. Each box contains the GO term identifier and description. Lines connecting GO terms represent ontology relationship (black lines indicate a regulatory relationship, and light blue lines indicate GO terms are a part of the term they connect to).

GO term enrichment analysis was performed on all significantly differentially expressed genes by examining term enrichment differences between upregulated and downregulated gene sets. Upregulated genes showed significant enrichment of biological processes for body morphogenesis, cuticle development, and chitin-based cuticle development (*P* ≤ 2.34 × 10^−3^ in all cases); significant molecular functions of structural constituent of cuticle, structural constituent of chitin-based cuticle, and structural molecule activity (*P* ≤ 3.41 × 10^−3^ in all cases); and significant cellular components involving the extracellular matrix (*P* ≤ 6.38 × 10^−4^ in all cases) (Figure 4). Downregulated genes showed significant enrichment of many biological processes related to immune and defense responses including the antimicrobial humoral response, response to bacteria, defense response to bacteria, and immune system process, among others (*P* ≤ 2.14 × 10^−3^ in all cases). Enriched cellular components include chorion, external encapsulating structure, and membrane-bounded organelles, among others (*P* ≤ 3.04 × 10^−2^ in all cases) (Figure 5). No significant molecular functions were enriched within the downregulated set of genes (the full list of significant GO terms can be found in Tables S3 and S4 in File S1).

## Discussion

Ecological specialization is a complex trait, involving numerous targets of selection that affect behavioral, physiological, and morphological phenotypes (Futuyma and Moreno 1988). As such, understanding the specific genetic basis of ecological adaptations to novel hosts is needed. In this study, we show the power of transcriptomics to reveal candidate genes involved in the evolution of host plant toxin resistance. By performing differential gene expression analysis on control and OA-exposed *D. sechellia* flies, we identified 104 genes that were differentially expressed, and in this set of 104 genes there was only one gene residing within the major effect QTL for OA resistance on chromosome 3R, *Osiris 6*. Prior studies have implicated *Osi6* as being involved in the derived OA resistance observed in *D. sechellia*. Andrade López *et al.* (2017) showed that global RNAi knockdown of *Osi6* in *D. melanogaster* adults led to increased OA susceptibility. Consistent with this finding, subsequent gene expression analyses revealed that *Osi6* expression was over 72 times higher in *D. sechellia* than its OA-susceptible sister species *D. simulans*. Further, a tissue-, environment-, and stage-specific downregulation of *Osi6* expression specifically in the adult salivary gland of *D. sechellia* was also discovered and RNAi knockdowns were consistent with this gene expression result (Andrade López *et al.* 2017). In addition to these findings in *D. sechellia*, a recent study focusing on an island population of *D. yakuba*, which recently evolved to specialize on *M. citrifolia*, also reported that the *Osiris* cluster may be involved, with a peak centered upstream of *Osi6* found to be among the strongest differentiation peaks in a population genomics scan between the specialist and mainland generalist populations of this species (Yassin *et al.* 2016). This study suggests that evolved plasticity of *Osi6* expression, increasing upon exposure to a high concentration of OA, may play an important role in OA resistance. This is consistent with RNAi knockdown of *Osi6* in *D. melanogaster*, suggesting the higher organism-wide expression of *Osi6* is favorable for OA resistance.

In addition to *Osi6*, we found five other genes that fall within other QTL for OA resistance. These genes include three genes without annotated function: *CG15740*, *CG1368*, and *CG9672*; a proneural gene, *l*(*1*)*sc* (*lethal of scute*), which plays an important regulatory role in both neurogenesis and dopaminergic neuron identity (Stagg *et al.* 2011); and *TweedleY* (*TwdlY*), a member of a *Drosophila* cuticle protein family that has been shown to alter body shape (Guan *et al.* 2006). These genes represent excellent candidates for further research into the molecular roles these genes play in *D. sechellia* and whether this is important for OA resistance. A recent study suggests that an evolved change in the catecholamine regulatory protein *Catsup* in *D. sechellia* combined with the presence of L-dopamine in *M. citrifolia* fruit has aided in the specialization of *D. sechellia* on its preferred host plant (Lavista-Llanos *et al.* 2014). The potential interaction between *l*(*1*)*sc* and this derived change in dopamine regulation in *D. sechellia* is possible and warrants further study. Additionally, our finding of differential *TwdlY* expression along with GO term enrichment for upregulated genes involved in cuticle development suggests a key role for a structurally-based mechanism of OA resistance acting through changes in the cuticle that could influence penetration of the insecticide.

GO term enrichment analyses revealed two main results: significantly upregulated genes are enriched for biological processes of body morphogenesis and cuticle development, and significantly downregulated genes are enriched for a response to bacteria and an antibacterial humoral response. It appears that exposure to OA results in a potentially weakened immune system and many immune genes are significantly downregulated (*e.g.*, *Def*, *edin*, *IM2*, *IM18*, and *IM23*). While the mechanism of OA toxicity is still unknown, the results of this study suggest that increased bacterial pathogenicity due to decreased immune function may contribute to lethality upon OA exposure in the wild, in addition to any direct effect of OA toxicity. A recent study showed that *D. sechellia* is unable to mount an immune response against parasitoids as lamellocyte-mediated encapsulation has been lost in this species (Salazar-Jaramillo *et al.* 2017). This apparently relaxed selection pressure on *D. sechellia* to maintain an immune response against parasitoids and microbes suggests that this case of host specialization allows for enemy-free space, a key selection pressure often involved in insect–host plant specificity (Jeffries and Lawton 1984). However, the ecological forces that contributed to host specialization in *D. sechellia* are still unknown. Further ecological, evolutionary, and genetic research is needed to determine if specialization was driven by interspecific competition, predation by parasitoids, another selective force, or some combination of these factors.

## Supplementary Material

Supplemental material is available online at www.g3journal.org/lookup/suppl/doi:10.1534/g3.117.300297/-/DC1.
