# Supplementary material for: Transcriptomic Analysis of Octanoic Acid Response in Drosophila sechellia Using RNA-Sequencing
Source: G3 (Bethesda). 2017 Oct 11;7(12):3867–73. doi: 10.1534/g3.117.300297 (PMC5714484; doi:10.1534/g3.117.300297)
Supplement: Supplementary file 1 [file 3867FileS1.pdf]

Supplementary Information

Supplemental Figure

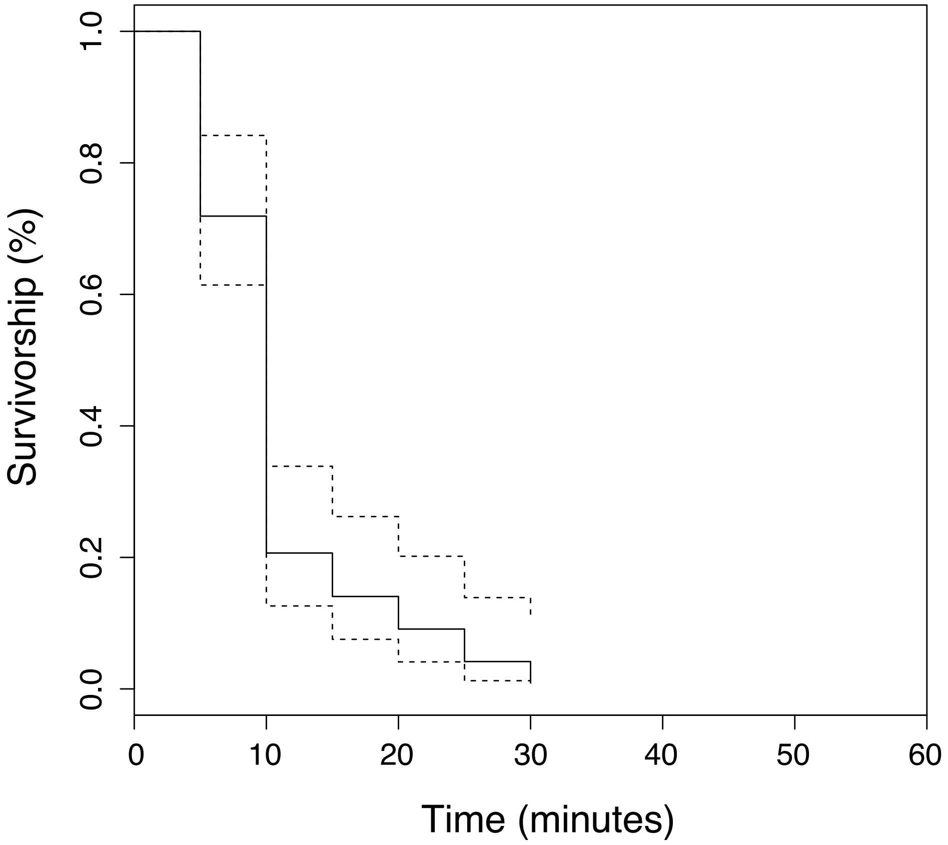

Supplemental Figure 1. The survival curve from an octanoic acid (OA) resistance assay is shown for *D. simulans* at a concentration of 0.7% OA. All flies assayed were knocked down within 30 minutes (n = 60). Dotted lines represent 95% confidence intervals from a Cox regression model (Cox 1972; Fox 2008).

Supplementary Tables

Supplementary Table 1. List of significantly differentially expressed genes upon exposure to 0.7% OA in *D. sechellia*.

***D. sechellia* gene**	***D. melanogaster* ortholog**	**Expression in control (FPKM)**	**Expression in OA (FPKM)**	**qvalue**
GM21567	*AttA*	299.311	16.8494	0.00369591
GM21465	*AttC*	619.82	166.673	0.00369591
GM10533	*Ccp84Ac*	0	2.68372	0.00369591
GM10910	*Ccp84Ag*	0	3.08253	0.00369591
CecA1	*CecA2*	872.378	80.2675	0.00369591
CecB	*CecB*	22.8332	0	0.0237434
GM10577	*CG10280*	14.9944	5.26221	0.03358
GM25738	*CG10407*	151.323	53.6545	0.00369591
GM13909	*CG10592*	160.741	77.6551	0.00369591
GM20285	*CG10814*	315.069	123.951	0.00369591
GM22074	*CG11137*	189.784	104.766	0.0209875
GM22449	*CG11425*	147.489	61.1621	0.00369591
GM24424	*CG13047*	0	2.23475	0.00369591
GM17353	*CG13083*	45.2505	15.3457	0.00369591
GM17352	*CG13084*	40.2714	14.2391	0.00369591
GM21372	*CG13159*	0	53.7365	0.00369591
GM13860	*CG13297*	0	5.75263	0.00369591
GM17654	*CG1368*	6.80424	26.6561	0.00369591
GM21466	*CG13749*	636.484	296.919	0.00974006
GM25236	*CG14147*	0	4.99158	0.00369591
GM22251	*CG14187*	82.0007	11.5423	0.00369591
GM17502	*CG14427*	6.27396	0	0.00369591
GM21046	*CG14752*	0	5.35846	0.00369591
GM20710	*CG14756*	0	6.23812	0.00369591
GM18840	*CG14915*	40.3444	0	0.00369591
GM13975	*CG15022*	0	5.86608	0.00369591
GM13933	*CG15213*	0	34.7585	0.0067546
GM13042	*CG15740*	0	5.15401	0.00369591
GM13954	*CG15876*	6.50185	0	0.00369591
GM14119	*CG16762*	15.5898	42.1094	0.00974006
GM10290	*CG16815*	2.89327	0	0.00369591
GM25218	*CG18628*	1142.77	562.283	0.00974006
GM26154	*CG31272*	25.4191	10.2968	0.0067546
GM15146	*CG31813*	0	17.9298	0.00369591

GM22817	*CG32548*	0	2.02356	0.00369591
GM14261	*CG3344*	96.06	56.7078	0.0315324
GM10110	*CG3348*	342.864	125.23	0.00369591
GM14909	*CG3819*	186.139	81.2924	0.00369591
GM18851	*CG43725*	62.5214	0	0.00369591
GM15682	*CG4377*	134.475	70.5272	0.0181747
GM17242	*CG5110*	114.28	241.177	0.00974006
GM14710	*CG5150*	149.74	66.5612	0.00369591
GM26400	*CG5791*	483.113	141.666	0.00369591
GM14989	*CG6885*	4.24459	0	0.00369591
GM14968	*CG7330*	0	2.25015	0.0067546
GM25710	*CG7542*	265.502	126.106	0.00369591
GM14841	*CG8562*	277.117	159.967	0.03358
GM14222	*CG9184*	0	28.1869	0.00369591
GM13371	*CG9672*	49.1188	21.1608	0.03358
GM25877	*CG9928*	1196.81	3129.7	0.00369591
GM11401	*Cht6*	0.866183	1.87078	0.0409988
GM15753	*Cht9*	57.1731	23.8922	0.00369591
GM25088	*Cp16*	390.056	37.6552	0.00369591
GM25085	*Cp18*	266.148	18.9007	0.00369591
GM25087	*Cp19*	925.42	157.152	0.00369591
GM13998	*Cpr64Ab*	0	3.03669	0.0126376
GM20557	*Def*	3106.25	889.394	0.00369591
GM21924	*DptB*	797.405	101.994	0.00369591
GM21566	*Dro*	9308.67	2612.53	0.00369591
GM14569	*Drs*	4226.32	1199.36	0.00369591
GM14560	*Drsl2*	15.1553	0	0.0126376
GM14562	*Drsl5*	2069.11	987.333	0.0067546
GM16160	*dunk*	18.5463	1.11548	0.0181747
GM13693	*e(r)*	257.404	489.369	0.0388071
GM10294	*E(spl)mgamma-HLH*	0	3.49195	0.00369591
GM25706	*edin*	301.734	35.7687	0.00369591
GM18842	*Fcp3C*	146.006	22.6801	0.00369591
GM12699	*Femcoat*	45.5622	6.85704	0.00369591
GM22021	*GNBP-like3*	518.503	15.923	0.00369591
GM24056	*Gnmt*	73.1719	31.5435	0.00369591
GM15526	*IM18*	3455.31	399.252	0.00369591
GM21868	*IM2*	6989.15	3509.61	0.00974006
GM19911	*IM23*	325.811	96.8803	0.00369591

GM14048	*ImpE2*	0	5.72309	0.00369591
GM19063	*l(1)sc*	0	2.04759	0.00369591
Mal-A7	*Mal-A7*	115.653	66.3171	0.0264442
GM19873	*MFS14*	109.109	47.0949	0.00369591
GM22138	*Neu2*	2.41556	0	0.00369591
GM23057	*Ntf-2*	217.94	416.855	0.03358
GM10877	*Osi15*	0	9.91204	0.00369591
GM10882	*Osi18*	0	3.00331	0.00369591
GM10883	*Osi19*	0	5.0218	0.00369591
GM10884	*Osi20*	0	9.19281	0.00369591
GM10867	*Osi6*	0	3.0258	0.00369591
GM10870	*Osi9*	0	2.27975	0.00369591
GM12960	*Peritrophin-15a*	5877.14	2900.75	0.00369591
GM24370	*PGRP-SB1*	322.452	91.6442	0.00369591
GM23808	*phu*	123.267	35.8208	0.00369591
GM10803	*RpII18*	205.263	389.02	0.0237434
GM15421	*Scp2*	145.75	280.363	0.0067546
GM21468	*Ser8*	183.584	90.5475	0.00369591
GM11667	*SmB*	264.06	150.007	0.0181747
GM25414	*SNCF*	30.5046	0	0.00369591
Sry-alpha	*Sry-alpha*	21.6102	1.79706	0.00369591
GM22735	*Tao*	94.7745	171.399	0.0290916
GM14935	*term*	15.7461	0.866257	0.0067546
TotA1	*TotA*	695.71	335.549	0.00369591
TotA1	*TotA*	702.268	331.185	0.00369591
TotC	*TotC*	190.273	63.1211	0.00369591
GM10719	*TwdlG*	0	3.35846	0.00369591
GM10177	*TwdlL*	0	3.15623	0.00369591
GM10330	*TwdlM*	0	11.3328	0.00369591
GM10739	*TwdlV*	0	2.06772	0.00369591
GM13463	*TwdlY*	0	3.27412	0.00369591

Supplementary Table 2. Significantly differentially expressed genes and transcripts upon 0.7% OA exposure in *D. sechellia* with no annotated ortholog in *D. melanogaster* or non-mRNAs.

D. sechellia gene or transcript	Gene Type	Predicted Function
RF00001	rRNA gene	ribosomal RNA
RF00002	rRNA gene	ribosomal RNA
RF00017	rRNA gene	ribosomal RNA
snoRNA:GM27480	snoRNA gene	small nucleolar RNA
snoRNA:GM27503	snoRNA gene	small nucleolar RNA
snoRNA:GM27504	snoRNA gene	small nucleolar RNA
snoRNA:GM27570	snoRNA gene	small nucleolar RNA
snRNA:U1:3	snRNA gene	small nuclear RNA
snRNA:U1:7	snRNA gene	small nuclear RNA
snRNA:U2:3	snRNA gene	small nuclear RNA
snRNA:U2:4	snRNA gene	small nuclear RNA
snRNA:U2:5	snRNA gene	small nuclear RNA
18SrRNA:GM27729	rRNA gene	ribosomal RNA
18SrRNA:GM27730	rRNA gene	ribosomal RNA
18SrRNA:GM27731	rRNA gene	ribosomal RNA
18SrRNA:GM27732	rRNA gene	ribosomal RNA
18SrRNA:GM27733	rRNA gene	ribosomal RNA
18SrRNA:GM27734	rRNA gene	ribosomal RNA
GM13041	NO ORTHOLOG	none
GM16569	NO ORTHOLOG	structural constituent of ribosome
GM18057	NO ORTHOLOG	serine-type endopeptidase activity
GM19428	NO ORTHOLOG	none
GM20514	NO ORTHOLOG	none
GM22086	NO ORTHOLOG	none
GM22087	NO ORTHOLOG	none
GM22462	NO ORTHOLOG	none
GM22463	NO ORTHOLOG	none
GM24426	NO ORTHOLOG	none

Supplementary Table 3. GO term enrichment of significantly upregulated genes.

GO Term	GO ID	Ontology	Fold Enrichment	*P*-value
body morphogenesis	GO:0010171	biological process	58.51	2.34E-03
chitin-based cuticle development	GO:0040003	biological process	16.54	7.37E-05
cuticle development	GO:0042335	biological process	11.97	8.63E-04
structural constituent of chitin-based cuticle	GO:0005214	molecular function	23.4	2.15E-06
structural constituent of cuticle	GO:0042302	molecular function	22.25	3.18E-06
structural molecule activity	GO:0005198	molecular function	7.34	3.41E-03
proteinaceous extracellular matrix	GO:0005578	cellular component	29.04	6.38E-04
extracellular matrix	GO:0031012	cellular component	15.49	2.91E-05

Supplementary Table 4. GO term enrichment of significantly downregulated genes.

GO Term	GO ID	Ontology	Fold Enrichment	*P*-value
antibacterial humoral response	GO:0019731	biological process	70.28	3.96E-08
defense response to Gram-positive bacterium	GO:0050830	biological process	47.02	2.99E-08
antimicrobial humoral response	GO:0019730	biological process	30.67	1.19E-05
humoral immune response	GO:0006959	biological process	28.54	1.13E-07
response to bacterium	GO:0009617	biological process	14.57	1.47E-08
defense response to other organism	GO:0098542	biological process	12.68	8.09E-08
immune response	GO:0006955	biological process	12.23	2.83E-05
response to external biotic stimulus	GO:0043207	biological process	12.16	5.29E-10
response to other organism	GO:0051707	biological process	12.16	5.29E-10
response to biotic stimulus	GO:0009607	biological process	12.16	5.29E-10
defense response to bacterium	GO:0042742	biological process	11.72	2.40E-04
defense response	GO:0006952	biological process	10.66	2.17E-08
immune system process	GO:0002376	biological process	7.7	2.01E-03
response to external stimulus	GO:0009605	biological process	4.47	3.67E-04
response to stress	GO:0006950	biological process	4.18	6.08E-05
multi-organism process	GO:0051704	biological process	3.36	2.14E-03
chorion	GO:0042600	cellular component	30.89	5.26E-04
external encapsulating structure	GO:0030312	cellular component	28.02	8.46E-04
extracellular space	GO:0005615	cellular component	7.26	1.03E-06
extracellular region	GO:0005576	cellular component	5.5	8.98E-10
extracellular region part	GO:0044421	cellular component	4.96	1.57E-04
intracellular part	GO:0044424	cellular component	0.34	5.20E-03
intracellular	GO:0005622	cellular component	0.33	3.68E-03
intracellular organelle	GO:0043229	cellular component	0.31	3.74E-02
organelle	GO:0043226	cellular component	0.3	2.74E-02
membrane-bounded organelle	GO:0043227	cellular component	0.24	3.04E-02
